# CXCL12 expression is an adverse predictor for disease recurrence in patients with metastatic non-seminomatous testicular germ cell tumors

**DOI:** 10.1186/s12885-019-5961-1

**Published:** 2019-08-14

**Authors:** Christian Daniel Fankhauser, Lisa Roth, Nico Christian Grossmann, Benedikt Kranzbühler, Daniel Eberli, Tullio Sulser, Holger Moch, Peter-Karl Bode, Joerg Beyer, Thomas Hermanns

**Affiliations:** 1 Department of Urology, University Hospital, University of Zurich, Zurich, Switzerland; 2 Department of Pathology of Molecular Pathology, University Hospital, University of Zurich, Zurich, Switzerland; 3 Department of Oncology, University Hospital, University of Bern, Bern, Switzerland

**Keywords:** Testicular germ cell tumor, Biomarkers, tumor, Prognosis

## Abstract

**Background:**

To validate the utility of the chemokine ligand 12 (CXCL12) as prognostic marker in patients with localized and metastatic germ cell tumors (GCT).

**Methods:**

CXCL12 expression was analyzed on a tissue microarray consisting of 750 tissue cores of different histological tumor components, Germ cell neoplasia in situ (GCNIS) and adjacent normal tissue of 263 testicular cancer patients using a semi-quantitative score. The association between CXCL12 expression and recurrence-free survival (RFS) as well as overall survival (OS) was assessed using Kaplan-Meier curves with log-rank tests.

**Results:**

CXCL12 expression was absent in all seminomas but was found in 52 of 99 (52.5%) non-seminomas. Follow-up was available for 260 patients of which 36 (13.8%) recurred. In patients with stage 1 non-seminoma GCT, CXCL12 expression was not associated with higher risk of disease recurrence (*p* = 0.270). In contrast, post chemotherapy RFS of patients with metastatic non-seminoma and positive CXCL12 expression was significantly shorter compared to CXCL12 negative patients (*p* = 0.003). OS differences were not statistically different between patients with CXCL12 positive or negative tumors for either localized or metastatic disease.

**Conclusions:**

CXCL12 is almost exclusively expressed in non-seminoma. Pure seminoma, GCNIS and adjacent normal testicular tissue are CXCL12 negative. Our analysis suggests that patients with metastatic disease and a CXCL12-positive non-seminoma are at higher risk for disease recurrence after first-line chemotherapy and might thus be candidates for more intensive treatment and/or closer follow-up.

## Background

Testicular germ cell tumors (GCT) represent the most common solid neoplasm of men in their third and fourth decade [1]. Seventy percent of all patients are initially diagnosed with localized disease (i.e. stage I disease) [2]. If the cancer is limited to the testicle, the therapy involves surgical removal of the affected testicle, followed by a strict surveillance program including regular blood tests, x-rays and computerized tomography [3, 4]. Despite the excellent prognosis of localized GCT, 15–30% of patients experience disease recurrence during surveillance [5]. An option to reduce the risk of recurrence is the administration of one cycle of adjuvant chemotherapy after orchiectomy, which reduces the risk of disease recurrence substantially [6]. Attempts have been made to stratify patients, who are at higher risk for disease recurrence based on different pathological characteristics (i.e. the size of the tumor and rete testis infiltration or the presence of lympho-vascular invasion and of embryonal carcinoma) [6–10]. However, the discovery of new markers that more reliably predict the risk of recurrence are of utmost importance.

Recently Gilbert et al. [11] were able to show that an increased expression of the chemokine ligand 12 (CXCL12) in the primary tumor was associated with a decreased relapse rate in patients with stage I non-seminoma. The authors concluded, that low CXCL12 expression might be a useful biomarker to identify patients who are at lower risk of recurrence and thus good candidates for active surveillance. However, those findings were somewhat surprising since CXCL12 is known as tumor promoting chemokine and high CXCL12 expression is actually associated with worse survival in most cancers [12]. The aims of our study were to externally validate the results of Gilbert et al. in patients with stage I disease and to extend the analysis to patients with metastatic GCT.

## Methods

A consecutive series of patients with surgically resected testicular GCT between 2000 and 2014 was evaluated. Patients with bilateral testicular tumors, extra-gonadal primary or missing follow-up data were excluded from the analysis. Clinical characteristics (age, Body-Mass-Index (BMI), histology of the primary tumor, staging characteristics according to the tumor, nodes and metastases system (TNM) of the American Joint Committee on Cancer (AJCC) and to the International Germ Cell Cancer Collaborative Group (IGCCCG) and blood-based tumor markers (i.e. alpha-fetoprotein (AFP), beta-human chorionic gonadotropin (b-hCG) and lactate dehydrogenase (LDH)) as well as follow-up information were extracted from the medical charts. The study was approved by the local ethics committee (KEK StV. 25–2008).

A tissue microarray (TMA) was constructed of formalin-fixed, paraffin-embedded tumor tissue of all included patients. If more than one tumor component was present in one patient all different GCT components were separately punched and included on the TMA. Tumors were classified by an experienced uropathologist (PKB) specialized in GCT pathology according to the 2016 WHO Classification [13]. Each tumor component was represented by two 0.6 mm diameter cores. The TMA was finally constructed with tissue cores of 263 GCT patients and included 99 Non-seminomas and 164 seminomas. The separately punched GCT components included: 422 seminomas, 172 embryonal carcinomas, 65 yolk sac tumors, 29 teratomas, 7 choriocarcinomas and 9 germ cell neoplasia in situ (GCNIS). Additionally, adjacent normal testicular tissue of 40 GCT patients was included.

**Fig. 1:**
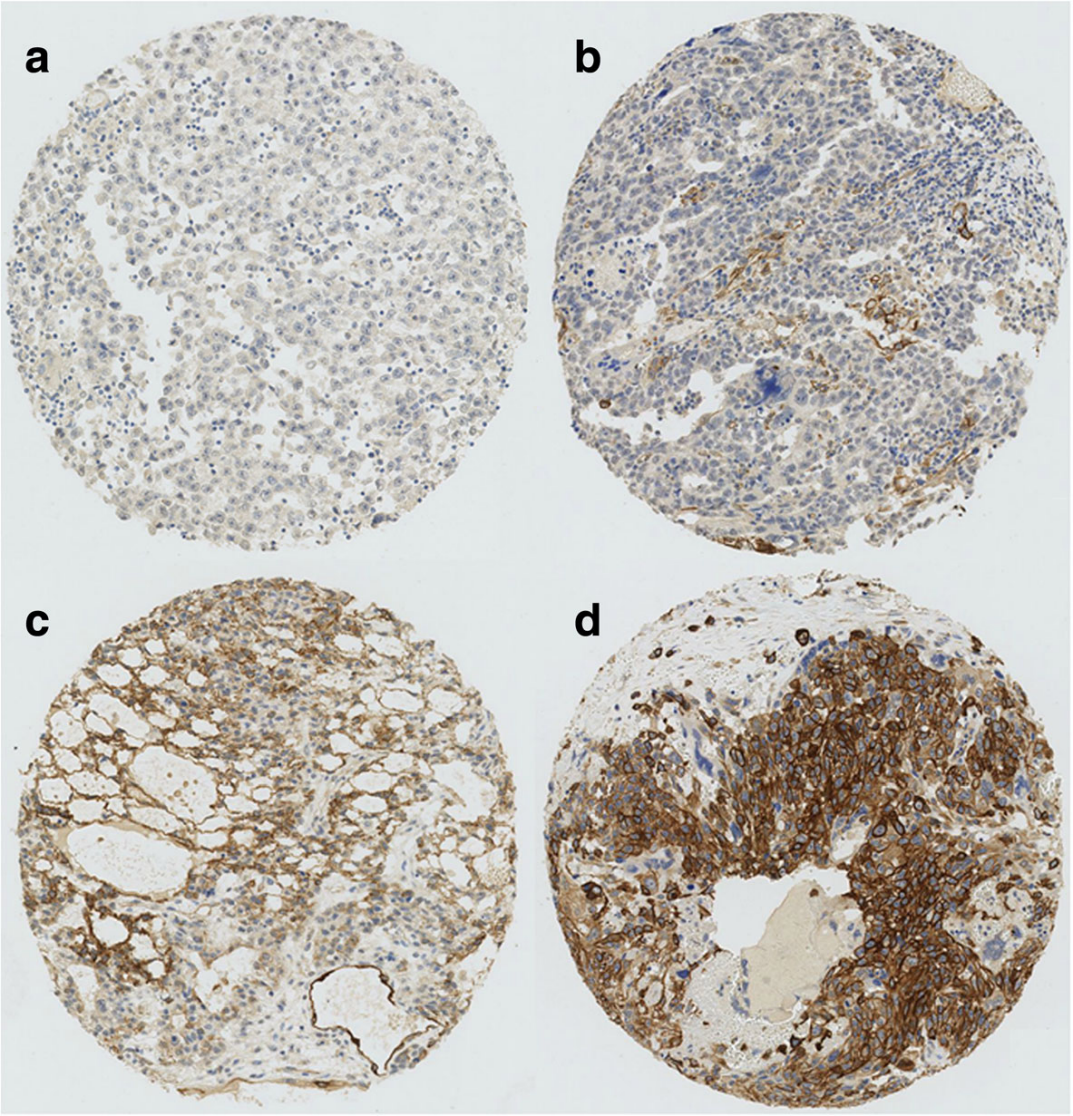
Examples of absent (**a**), weak (**b**) moderate (**c**) and strong (**d**) CXCL12 in seminoma (**a**), embryonal carcinoma (**b** & **d**) and yolk sac (**c**) tumor components

Sections of 3 μm thickness of the TMA blocks were mounted on glass slides (SuperFrost Plus; Menzel, Braunschweig, Germany), deparaffinized, rehydrated and stained with hematoxylin and eosin using standard histological techniques. CXCL12 immunohistochemistry was performed using the monoclonal CXCL12 antibody (Antibody 79018, 1:100; R&D Systems Minneapolis MN, USA). Peripheral neuronal tissue served as internal positive control for CXCL12 staining. The stained slides were digitalized and evaluated using imaging software (Spot browser, Alphelys, Plaisir, France). Two investigators (CDF, PKB) evaluated the CXCL12 staining of each spot on the TMA. A patient’s tumor was classified as being positive if any of its components showed either moderate or strong CXCL12 expression and negative if CXCL12 expression was either absent or weak (Fig. 1).

Recurrence-free survival (RFS) and overall survival (OS) were calculated from time of diagnosis to first recurrence after initial management or death respectively. RFS and OS of patients with CXCL12 positive and negative tumors were compared using Kaplan-Meier curves and the log-rank test. Statistical analysis was performed using IBM SPSS Statistics (Version 21.0, Armonk, New York, USA: IBM Corp.). The results for continuous normally distributed variables are expressed as means ± standard deviation (SD). Continuous non-normally distributed variables are presented as median and interquartile ranges (IQR). The results for categorical variables are presented as percentage. A *p*-value of < 0.05 was considered significant. All statistical tests were two-sided.

## Results

The mean age at diagnosis was 26 years and patients were followed for a median of 64 months (Table 1). At initial diagnosis 190 of 263 patients (72%), were classified as stage I, 4 patients (2%) as stage IS, 35 patients (13%) as stage II and 34 patients (13%) as stage III. Of 132 patients with stage I seminoma, adjuvant chemotherapy with 1 cycle of carboplatin was administered to 70 patients (53%) and adjuvant radiotherapy to 6 patients (5%). Of 58 patients with stage I non-seminoma, adjuvant chemotherapy with 1 cycle of bleomycin, etoposide and cisplatin (BEP) was administered to 13 patients (22%). Of all 73 patients with metastases, 56 (76%) were classified as good risk, 13 (18%) as intermediate risk and 4 (6%) as poor risk according to the IGCCCG classification. All metastatic patients were treated with cisplatin based chemotherapy, most commonly with BEP (83%). Seven patients underwent retroperitoneal lymph node dissection due to post-chemotherapy residual masses.

**Table 1:** Baselines characteristics

	*N* = 263
Age (years) (±SD)	26.4 (8.9)
Seminoma	164 (62%)
Non-Seminoma or mixed GCT	99 (38%)
Clinical Stage (AJCC)	
-Stadium I	190 (72%)
-Stadium IS	4 (2%)
-Stadium II	35 (13%)
-Stadium III	34 (13%)
IGCCCG risk groups	
-Good risk	56 (21%)
-Intermediate risk	13 (5%)
-Poor risk	4 (2%)

*AJCC* American Joint Committee on Cancer, *BMI* Body-Mass-Index (kg/cm2), *IGCCCG* International Germ Cell Cancer Collaborative Group, *IQR* Interquartile Range, *SD* Standard deviation, *TNM* tumor, nodes and metastases system of cancer staging

**Fig. 2:**
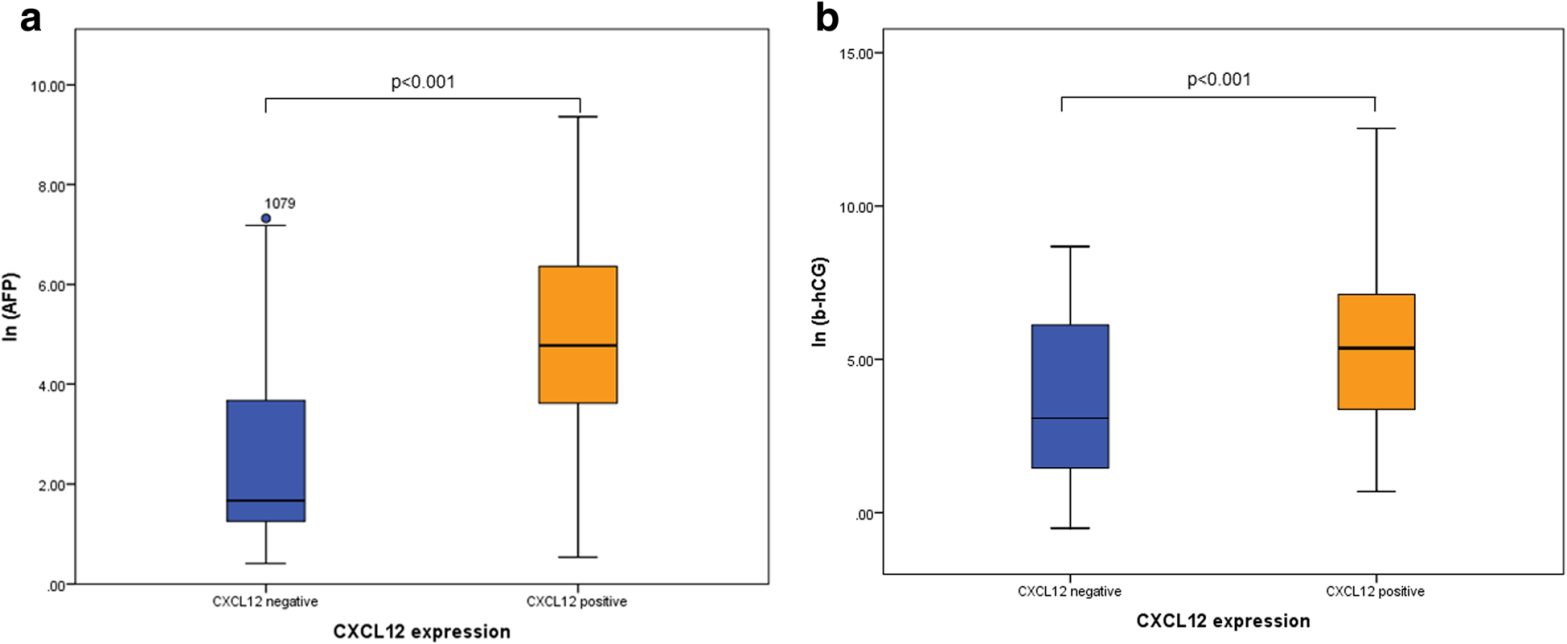
Boxplots comparing AFP (**a**) and b-hCG (**b**) levels before orchiectomy between patients with negative and positive CXCL12 expression

CXCL12 expression was negative in normal tissue, GNCIS and seminoma tissue (Table 2). In patients with non-seminoma, CXCL12 expression was found in 52 patients (52.5%). Non-seminoma patients with CXCL12 expression in their primary tumor had significantly higher pre-orchiectomy AFP values (118.7 μg/l vs. 5.3 μg/l, *p* < 0.001) and b-HCG values (152.0 U/l vs. 8.2 U/l, p < 0.001) compared to CXCL12 negative patients (Fig. 2).

**Table 2:** CXL12 expression on single core level and patient level

Single Cores	Semi-quantitative Score			
	negative	weak	moderate	strong
Seminoma (*n* = 422)	421 (99.8%)	0 (0.0%)	0 (0.0%)	1 (0.2%)
Embryonal Carcinoma (*n* = 172)	123 (70.7%)	0 (0.0%)	8 (4.6%)	43 (24.7%)
Teratoma (*n* = 29)	19 (59.3%)	0 (0.0%)	2 (6.3%)	11 (34.4%)
Yolk sack Tumor (*n* = 65)	5 (7.6%)	1 (1.5%)	11 (16.7%)	49 (74.2%)
Chorion Carcinoma (*n* = 7)	0 (0.0%)	0 (0.0%)	1 (14.3%)	6 (85.7%)
Patient Level	Dichotomized			
	Negative		Positive	
Non Seminoma or mixed GCT (*n* = 99)	47 (47.5%)		52 (52.5%)	
Pure Seminoma (*n* = 164)	164 (100%)		0 (0.0%)	
Normal tissue (*n* = 40)	40 (100%)		0 (0.0%)	
GCNIS (*n* = 9)	9 (100%)		0 (0.0%)	

Abbreviation: *GCT* germ cell tumor, *GCNIS* Germ cell neoplasia in situ

**Fig. 3:**
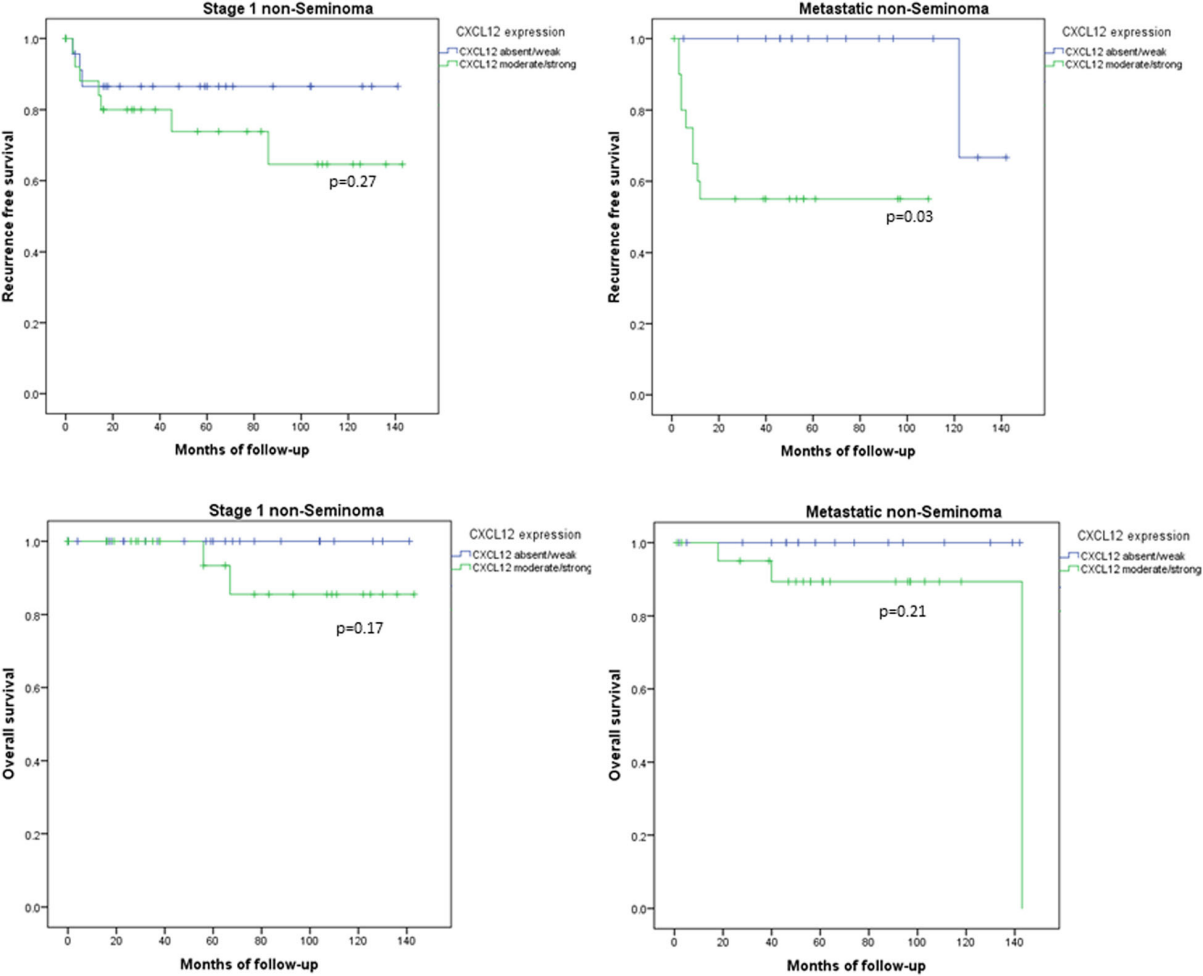
Kaplan Meyer curves for recurrence free (top row) and overall survival (lower row) stratified by CXCL12 expression. In stage 1 non-seminoma CXCL12 expression did neither predict recurrence free (top left corner) nor overall survival (lower right corner). In metastatic non-seminoma CXCL12 expression predicted recurrence free (top right) but not overall survival (lower right)

In patients with localized non-seminoma, CXCL12 expression was not significantly associated with time to disease recurrence (*p* = 0.27) or time to death (*p* = 0.17) (Fig. 3). Also after controlling for adjuvant chemotherapy, no significant association between CXCL12 expression and time to recurrence was observed (*p* = 0.4). There was even a trend for a shorter time to recurrence in patients with high CXCL12 expression.

In patients with metastatic non-seminoma CXCL12 expression was not associated with worse IGCCCG risk groups (*p* = 0.66). In contrast, patients with metastatic non-seminoma and higher CXCL12 expression showed a significantly shorter time to disease recurrence (*p* = 0.03), whereas time to death was not significantly different (*p* = 0.21) compared to CXCL12 negative patients.

## Discussion

In this study CXCL12 expression was observed in a large number of patients with non-seminoma but was absent in normal tissue, GCNIS or seminoma. We were not able to confirm the results by Gilbert and colleagues and even found a trend for a higher risk of disease recurrence in patients with high CXCL12 expression. Similarly to our results in stage I disease, CXCL12 expression was associated with a higher risk for disease recurrence in patients with metastatic non-seminoma after first line chemotherapy.

The chemokine CXCL12 has important physiological roles in embryonal development, vascular proliferation, hematopoiesis, and inflammation by interaction with different receptors such as the CXC chemokine receptor 4 (CXCR4) [14–16]. CXCL12/CXCR4 interaction is also known to be involved in tumor-promoting mechanisms which result in increased proliferation, invasion, adhesion, angiogenesis and decreased apoptosis [12]. High CXCL12 expression is known to be associated with worse survival in patients with different types of solid cancers [12].

The role of CXCL12 in GCT is controversial. In contrast to most solid cancers Gilbert et al. found that CXCL12 expression is a protective factor in patients with stage 1 non-seminoma [12]. However, in our analysis we were not able to confirm these findings of Gilbert and our analyses suggest that CXCL12 might even be a risk factor and not a protective factor in stage 1 non-seminoma. Although both studies used the same antibody, our TMA based approach might have missed the heterogeneity of CXCL12 expression. As a conclusion of those contradictory results we currently discourage clinicians to use CXCL12 for clinical decision making until further studies clarify the role of CXCL12 in in stage 1 non-seminoma.

For metastatic GCTs, the IGCCCG classification is currently recommended to counsel patients with metastatic GCT regarding their risk of disease recurrence and survival and to guide treatment for these patients [17]. This classification is based on the histological subtypes (i.e. seminoma vs. non-seminoma), the location of the primary tumor and of the metastases and the tumor markers levels. Our results suggest that CXCL12 expression in the primary tumor may improve risk stratification in patients with metastatic GCTs. Patients with metastatic disease and CXCL12 positive primary tumors may need more intense first-line chemotherapy and/or at least a closer post-chemotherapy follow-up.

The present analysis is limited by its retrospective design, single center approach and relatively small sample size. A further limitation of our study is that we were not able to perform a meaningful multivariate cox regression analysis due to the limited number of events. Additionally, multiple testing may lead to type I errors, which might overestimate the described associations between CXCL12 expression and oncologic outcomes. Thus, our analyses should rather be considered exploratory. A larger dataset including lymph vascular invasion status or IGCCCG classification is needed to reliably assess a potential independent prognostic impact of CXCL12 expression in localised and metastatic disease respectively. Especially the planned IGCCCG update might be an opportunity to elucidate whether CXCL12 would ultimately possess the potential to predict oncologic outcomes besides the IGCCCG classification [18].

## Conclusion

CXCL12 is exclusively expressed in non-seminoma and absent in seminoma, normal tissue or GCNIS. We were not able to show an association between CXCL12 and lower recurrence rates in stage I non-seminoma patients. However, positive CXCL12 expression was associated with shorter RFS after first-line chemotherapy in patients with metastatic GCT and thus might help to identify patients at a higher risk for disease recurrence.

## Data Availability

The datasets generated during and/or analyzed during the current study are available from the corresponding author on reasonable request.

## Abbreviations

AFP: Alpha-fetoprotein
AJCC: American Joint Committee on Cancer
b-hCG: beta-human chorionic gonadotropin
CXCL12: Chemokine ligand 12
GCNIS: Germ cell neoplasia in situ
GCT: Testicular germ cell tumors
IGCCCG: International Germ Cell Cancer Collaborative Group
IQR: Interquartile ranges
LDH: Lactate dehydrogenase
OS: Overall survival
RFS: Recurrence-free survival
SD: Standard deviation
TMA: Tissue microarray
TNM: Tumor, nodes and metastases system
